# Cell-Mediated Release of Nanoparticles as a Preferential Option for Future Treatment of Melanoma

**DOI:** 10.3390/cancers12071771

**Published:** 2020-07-02

**Authors:** Anastasia Chillà, Francesca Margheri, Alessio Biagioni, Tommaso Del Rosso, Gabriella Fibbi, Mario Del Rosso, Anna Laurenzana

**Affiliations:** 1 Department of Experimental and Clinical Biomedical Sciences School of Health Sciences, University of Florence-Viale G.B. Morgagni, 50–50134 Florence, Italy; anastasia.chilla@unifi.it (A.C.); francesca.margheri@unifi.it (F.M.); alessio.biagioni@unifi.it (A.B.); fibbi@unifi.it (G.F.); 2 Department of Physics, Pontifical Catholic University of Rio de Janeiro, 22451-900 Rio de Janeiro-RJ, Brazil; tommaso@puc-rio.br

**Keywords:** melanoma, nanoparticles, cell therapy, tumor microenvironment

## Abstract

Targeted and immune therapies have unquestionably improved the prognosis of melanoma patients. However the treatment of this neoplasm still requires approaches with a higher therapeutic index, in order to reduce shortcomings related to toxic effects and aspecific targeting. This means developing therapeutic tools derived with high affinity molecules for tumor components differentially expressed in melanoma cells with respect to their normal counterpart. Nanomedicine has sought to address this problem owing to the high modulability of nanoparticles. This approach exploits not only the enhanced permeability and retention effect typical of the tumor microenvironment (passive targeting), but also the use of specific “molecular antennas” that recognize some tumor-overexpressed molecules (active targeting). This line of research has given rise to the so-called “smart nanoparticles,” some of which have already passed the preclinical phase and are under clinical trials in melanoma patients. To further improve nanoparticles partition within tumors, for some years now a line of thought is exploiting the molecular systems that regulate the innate tumor-homing activity of platelets, granulocytes, monocytes/macrophages, stem cells, endothelial-colony-forming cells, and red blood cells loaded with nanoparticles. This new vision springs from the results obtained with some of these cells in regenerative medicine, an approach called “cell therapy.” This review takes into consideration the advantages of cell therapy as the only one capable of overcoming the limits of targeting imposed by the increased interstitial pressure of tumors.

## 1. Introduction

Considered until a few years ago to be a rare neoplasm, today melanoma shows a constant growth of incidence all over the world and numerous studies suggest that it has even doubled in the last 10 years [1,2]. Worldwide, cutaneous melanoma is estimated to have reached 100,000 new cases a year [1]. Despite the increase in the survival rate after 5 years from diagnosis, the lack of “definitive” treatments, besides the surgical ones for the initial forms of non-metastatic melanoma, has stimulated new therapeutic approaches, taking advantage of the most recent acquisitions provided by biomedical research, an issue extensively taken into consideration in other excellent reviews [3,4,5]. Among the new therapeutic approaches, treatments exploiting the properties of nanoparticles (NPs) hold meaningful perspectives to overcome drawbacks of classical chemotherapy, targeted therapy, topical therapy, and immunotherapies [3,4,5]. Adoptive cell transfer (ACT) appears to be a promising therapeutic option for patients with advanced metastatic melanoma, demonstrating a lasting and complete response in 24% of patients, with a median survival of over 3 years [6]. However, even with new immunotherapy, adverse effects (AE) can be very important and often require immunosuppression to be controlled [7].

Indeed, NPs may function as therapeutic and diagnostic devices, providing a “theranostic” multi-modal approach to diagnosis and therapy of primary and metastatic tumors, including melanoma [8,9,10]. Taking advantage of their nanometer scale, composition, and tunable chemical properties, NPs have been used for diagnosis and treatment of tumors from the beginning of the 1980s, and today the literature on tumor treatments with nano-based therapeutics amounts to over 24,000 contributions. NPs are nano-scaled objects ranging from 1 nm to even 500 nm and are composed of organic and inorganic compounds to be used as such or as carrier platforms for therapeutic molecules. Organic nanocarriers include phospholipid micelles, vesicles, multilamellar liposomes, dendrimers, and carbon nanotubes, while inorganic ones include quantum dots (composed by atoms of group II and group VI of the periodic table), gold NPs, silica (mesoporous) NPs, and superparamagnetic iron-oxide-NPs (SPIONs), all excellently reviewed in [11]. Nanotechnology and nanomedicine applied to tumor therapy went hand in hand since the first descriptions of NPs, exploiting passive targeting based on the enhanced permeability and retention effect (EPR), or by conjugating biorecognition molecules on their surface (active targeting). This last approach is based on the combined effects of passive EPR and active recognition of cancer molecular markers. Some of these advanced class NP-based platforms, able to deliver chemotherapeutics or photosensitizing agents (biodegradable NPs with a controlled drug release) to specific tumors and currently known as “smart nanoparticles,” have been approved by the FDA and are used in the treatment of various types of cancer, including metastatic uveal malignant melanoma (Marqibo) [2,12]. However, even with the new formulations of smart NPs, tumor targeting still remains the main problem. There is a considerable intra and inter-individual heterogeneity in EPR and EPR/Smart-NPs-mediated tumor targeting, which accounts for heterogeneous outcomes of several clinical trials. Such drawbacks have elicited efforts to identify possible means to increase patients response by therapeutic enhancement of EPR, as recently reviewed [13]. To overcome these drawbacks a parallel line of research is exploiting the innate or induced tumor tropism of living circulating cells carrying a NP payload to enhance tumor availability of theranostic NPs. These are a sort of “cellular cyborgs,” prepared from normal circulating cells, with an innate tropism toward tumors [14]. The cells most used as vehicles of NPs in cancer therapy both for their ability to distribute themselves in all the compartments of the vascular bed and for their innate properties of storage in the tumor masses, are: platelets, polymorphonuclear neutrophils, macrophages, and a series of stem cells, such as neural stem cells (NSCs), induced pluripotent stem cells (iPSCs), mesenchymal stem cells (MSCs), and a particular subpopulation of endothelial progenitors called ECFCs (endothelial colony-forming cells) [14,15,16]. In this review we will consider the “tug of war game” that any NP-based therapeutic and NP-loaded cell must sustain in order to locate within tumors. This process exploits a series of specific mechanisms to overcome tumor interstitial pressure (TIP) and to activate the release of NPs payload for a satisfactory destruction of primary tumors and their metastases.

## 2. TIP and EPR, Two Opposite Physical Forces

### 2.1. TIP and Fluid Stress

Under normal conditions the formation of the interstitial fluid is regulated by the so-called Starling equation, formulated in 1896, that illustrates the role of hydrostatic and oncotic pressures (the so-called Starling Forces) in the movement of fluids through capillary membranes. According to the Starling equation, the net filtration is given by the difference between capillary hydrostatic pressure + interstitial oncotic pressure (net filtration pressure) − interstitial hydrostatic pressure + capillary oncotic pressure (net resorption pressure). Normally the net filtration pressure prevails over the net reabsorption pressure of about 4 mm Hg, which would cause a continuous accumulation of liquid in the interstitial tissue. This does not happen because of the drainage of the lymphatic system, which conveys the excess of liquid into the venous circulation [17].

Tumor cells proliferate owing to their unlocking from the mechanisms that regulate the cell cycle and apoptosis. The pre-existing vessels become insufficient to nourish the increase in tissue mass and many parts of the tumor become ischemic, leading to the recruitment of macrophages and to proliferation of interstice stromal cells, such as cancer-associated fibroblasts (CAFs). Both macrophages and cancer cells increase the production of angiotropic cytokines, vascular endothelial growth factor-A (VEGF-A), platelet-derived growth factor (PDGF), and transforming growth factor-β TGF-β [18]. Under their influence, the tumor vessels proliferate to meet the increased oxygen demand of the tumor mass. However, the new vessels have a tortuous appearance, with a lack of a muscular layer and pericytes, narrowings and dilations, and the presence of large fenestrations, which can reach a diameter of about 5 µm (the majority of these fenestrations however has a diameter that varies between 1 and 100 nm) [19]. All these features make the tumor vessels hyper-permeable, with consequent increase of liquid and proteins in the tumor interstice, increase of the TIP and reduction of the intravascular hydrostatic pressure and therefore of the pressure gradient that in normal conditions determines a net filtration rate from the vessels to the tumor mass.

Lymphangiogenesis is also impaired, owing to the strong production of factors that stimulate the tumultuous growth of the lymphatic vessels (VEGF-C and VEGF-D) [20,21]. The lymphatic vessels form a very dense network around the tumor, but are tortuous and immature on the periphery and are deficient in swallow-nest valves, favoring the stagnation of the lymph [20,21]. As a result, both vascular and lymphatic changes promote a fluid-dependent increase in TIP.

### 2.2. TIP and Solid Stress

The growing solid mass of a vascularized organ causes vascular stress through the compression of the vessels (arterial, venous, and lymphatic). In tumors, the solid mass is represented by both tumor cells and the cellular and molecular components of the tumor microenvironment that increase stiffness of the cancer tissue (CAFs, macrophages, endothelial cells, collagen fibers, glycosaminiglycans (GAGs) including hyaluronic acid) [22]. As a consequence, the tumor tissue is more rigid and thick and exerts greater pressure on the vascular and intratumor lymphatic network [18] thus increasing TIP.

### 2.3. Consequences of TIP Increase on Circulation Times of Nanomedicines and EPR Effect

Conventional chemotherapy is based on the use of low molecular weight substances usually less than 1000 Da [23], with a short blood half-life and significant accumulation in non-target organs, thus causing serious side effects. The increase in molecular weight, obtained for example through encapsulation of chemotherapeutics in liposomes, extends the half-life from 5–10 min to 2–3 days [24], an effect also enhanced by the stealthy surface modification with polyethyleneglycol (PEG) [25]. The prolongation of the permanence in the circulation allows its accumulation in the tumor masses through the EPR effect, originally described in 1986 [26]. Indeed, all nanoparticles target cancer by simple accumulation and entrapment in tumors by EPR (passive targeting). EPR is caused by leaky angiogenetic vessels and poor lymphatic drainage and has been used to explain why macromolecules and nanoparticles are found at higher ratios in tumors compared to normal tissues. Grafting recognition molecules on the nanocarrier surface is called “active targeting” and is aimed at increasing the uptake of NPs by tumor cells. Nevertheless, elevated TIP impacts negatively on the treatment with conventional chemotherapy and targeted therapy, as well as with passive and active targeting with NPs, through many mechanisms. First, the transport process through the endothelial barrier into the interstitial space depends primarily on pressure gradients. If the pressure within the vessel is higher than TIP both large and small therapeutics are filtered and enter the interstitial space through convection. Instead, when TIP increases the gradient becomes inefficient and therapeutics laboriously and slowly enter within the tumors by diffusion. This is a very less efficient process, and therapeutic substances may also flow backward in the systemic circulation without reaching an useful therapeutic concentration within the tumor mass [27]. However, once entered in the interstitial space of tumors it is likely that NPs (maximally those hosted within micelles or liposomes) may remain for long times at the boundary between vessels and tumor because of the weakness of flow exchange between the compartments. A further mechanism is related to solid stress of tumors, which causes compression of the tumor vessels thereby increasing vascular resistance and TIP [28,29]. All these evidences have stimulated a line of research aimed at exploiting pharmacological and biological agents able to target the main mechanisms that control the increase of TIP as an adjuvant therapy associated to classical chemotherapy and NP therapy of tumors (exhaustively reviewed in refs. [13,18]).

In recent years it has been realized that the EPR effect is very heterogeneous with temporal variations between different individuals, in the same individual and in the same type of tumor. The differences exist not only between patients and mouse models, but also between primary tumor and metastasis in the same patient [30,31]. This may justify the differences found between preclinical results and therapeutic success with new treatments based on the use of NPs [32], although some retrospective studies have provided a pessimistic view on passive and active tumor targeting methods [30,33,34]. Other studies have instead shown that the concentrations reached in tumors by nanotherapeutics, even if low, are similar to those reached by conventional chemotherapy [35,36,37,38], thus justifying the therapeutic successes with NP-based specialties approved for clinical use. A clarification on the heterogeneity of the EPR effect and an intensification of studies aimed at obtaining a decrease in TIP to enhance the EPR effect are certainly necessary [39].

## 3. Cell-Mediated Delivery of Nanoparticles as a Means to Overcome TIP-Dependent Restraints of NP-Based Therapeutic Efficiency

**Figure 1:**
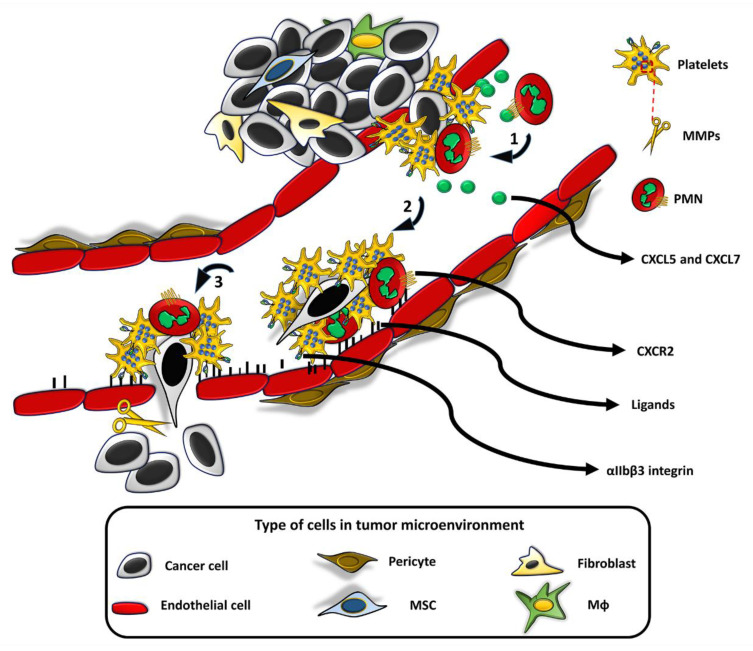
Role of platelets and PMN in metastatic tumor cell dissemination. Tumor cells detach from the primary tumor and invade the circulation. Upon their very first appearance in the blood, tumor cells are recognized as a “thrombogenous surface” thereby activating platelets that encircle and encase tumor cells. Activated platelets release substances stored in their alpha granules, including chemokines CXCL5 and CXCL7 (green spheres in the figure) that interact with CXCR2 receptor of neutrophils which are in turn recruited in the mixed cell agglomerate (**phase 1** in the figure). The binding of neutrophils to platelets is mediated by interaction of the sialyl-Lewis X of neutrophils with P-selectin released by alpha granules and exposed on the platelet surface (41, 46–50). While floating in the circulation (**phase 2** of the figure), a complex mixture of growth factors from platelets induces the shift of tumor cell phenotype from an epithelial to a mesenchymal-like and the mixed agglomerate begins to slow down and to roll on the endothelial surface, exploiting the interaction of P-selectin with PSGL-1 (P-selectin glycoprotein ligand-1) expressed on all the involved cells, including endothelial cells. Multiple interactions are required in this phase (46–50) (various ligands and the many molecules involved on the endothelial side are represented as small black rectangles that project into the blood vessel). The definitive adhesion and stop at the metastatic site (**phase 3** of the figure) occurs between neutrophil integrins, platelet α2/β3-integrin, and P-selectin, with various cell-adhesion molecules (CAM) expressed on endothelial cells. Cell extravasation is facilitated by P2Y2, a purinergic receptor expressed by platelets and endothelial cells: adenine nucleotides released by platelets interact with P2Y2 and “open” the endothelial barrier. Platelets matrix metallo proteinases (MMPs), tumor cell MMPs, and cell-associated fibrinolytic system degrade the extracellular matrix for an irreversible homing of metastatic cells.

**Figure 2:**
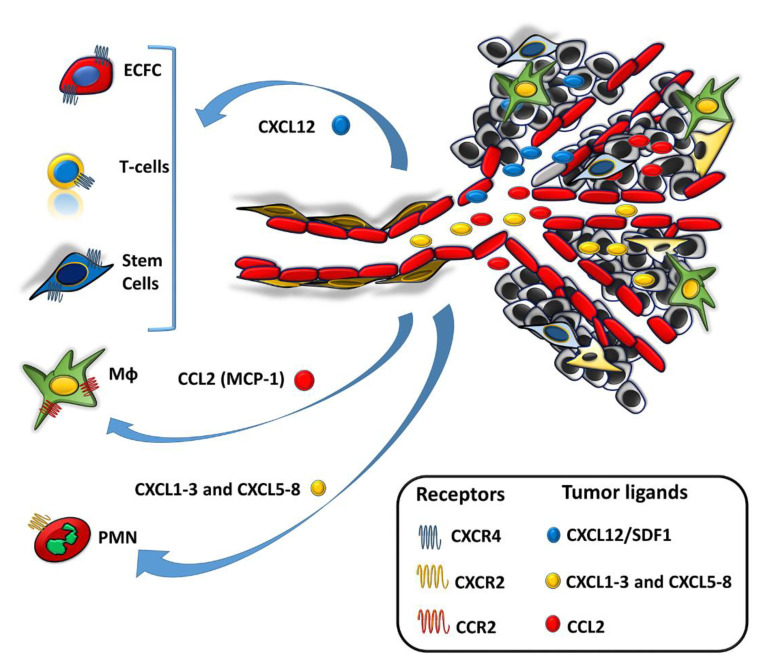
The main chemokine/chemokine-receptor axes involved in cell recruitment at the tumor site. Both tumor cells and cells of the tumor microenvironment [endothelial cell, monocytes/Mϕ (tumor associated macrophages, TAM), mesenchymal stem cells (MSCs), fibroblasts (cancer associated fibroblasts, CAFs)] release chemokines that recruit circulating cells into the tumor mass. Such chemokines interact with chemokine receptors expressed on circulating cells. The CXCL12 chemokine (SDF-1, stromal derived growth factor) recruits cells expressing the CXCR4 receptor: all the stem cells, [neural stem cells, induced pluripotent stem cells (iPSCs), mesenchymal stem cells (MSCs)], T lymphocytes, and endothelial colony-forming cells (ECFCs). The CCL2 chemokine [monocyte chemoattractant protein-1 (MCP-1)] recruits monocytes/Mϕ that express the CCR2 receptor. The CXCL1-3 and CXCL5-8 chemokines recruit neutrophils that express the CXCR2 receptor. For the cell types depicted in the tumor microenvironment, please refer to the tumor components in Figure 1.

Many cell types have an innate property of entering into tumors, regardless of EPR and other local conditions leading to an increase in TIP [14,15,16]. To carry out this task, the cells must express molecules that allow them to migrate and stop at the tumor endothelium in response to chemotactic substances synthesized by the cancer cells: specific receptors for cancer-produced chemo-attractants, intracellular contractile structures that allow them to activate the typical contraction-relaxation cycles of the cell body required for the “grip-and-go” process of cell movement. All these cells must also produce proteolytic enzymes necessary to open a way in the extracellular matrix of the tumor. On the basis of such properties, the following cells have been used for the release of their NPs payload, or for exploitation of their thermotransductive or molecular imaging power once within the tumors (Figure 1 and Figure 2).

### 3.1. Platelets

Recent research has proven the effectiveness of doxorubicin-loaded platelets as drug delivery vehicles in lymphoma [40], thus highlighting the theoretical use of platelets as a drug delivery system. Platelet activation is amplified in many cancers and platelets are found within the tumor vasculature [41,42,43,44,45]. There is evidence that the activation of platelets and the coagulation system promote the metastatic progression of cancer and are important in the development of blood metastases [45]. Tumor cells enter the blood stream, bind and activate platelets and leukocytes, these complexes arrest at the vessel wall enabling extravasation of tumor cells and their survival and proliferation within target tissues of metastasis [46,47,48,49]. Rolling of circulating tumor cells to the vessel wall, and formation of aggregates with platelets and leukocytes are mediated by selectins, a family of transmembrane cell adhesion molecules expressed by platelets, endothelial cells, and leukocytes. Metastasis studies indicate that platelets use selectins to support initial transient tumor cell interaction with the endothelium, similarly to what happens during leukocytes recruitment in inflammation. Integrins mediate the shift from selectin-dependent tumor cell rolling on the endothelium to firm arrest (Figure 1). The main platelet integrin involved in this process is integrin αIIbβ3 [50]. These events happen at sites of vessel wall lesions where platelet and tumor cells complexes encounter denuded areas of subendothelial matrix: they attach to these sites and become activated. Such endothelial microlesions are possibly associated to the so-called metastatic niche or be generated through leukocyte- or tumor cell-induced endothelial retraction [41].

Finally, platelets contain matrix-metallo-proteinases (MMPs) that are released upon platelet activation and may be exploited to open a path to their infiltration in tumors [51].

All the foregoing considerations authorize to consider platelets as legitimate nanotherapeutic carriers in tumors with the primary scope of targeting the heteroaggregates between platelets and metastatic cells. Indeed, platelets may be loaded with nano-scale materials [40], as supported also by their phagocytosis properties, already demonstrated in a work of many decades ago [52]. Melanoma is not an exception and recent studies have highlighted the role and prognostic significance of platelets in the metastatic propensity of human melanoma [53,54,55,56]. A recent paper has shown the possibility to target an experimental melanoma with red blood cells and platelet membrane-coated (“ghost-cell” strategy) gold nanostars containing curcumin [57]. RBC membrane coating provided self-antigens thus evading clearance by macrophages, while platelet membrane coating provided targetability to cancer cells. Controlled curcumin release was obtained under near-infrared irradiation.

Platelets have a life span of 7 to 12 days, cannot proliferate and are rather difficult to prepare in a purified form. However, given these limitations, platelets must be taken in serious consideration for their unique property to target tumor metastasis (Table 1).

### 3.2. Neutrophils

In experimental cancer models, neutrophils have been identified in almost every phase of tumor progression [58,59,60]. Overall, neutrophils infiltration directly correlates with poor clinical outcomes in several cancers, including melanoma [61,62,63,64,65], where a high neutrophil-to-lymphocyte ratio (NLR) indicates a poor outcome [66] suggesting that neutrophils and NLR could be considered potential prognostic markers for melanoma patients. The release of neutrophils from bone marrow into the circulation mainly depends on the interplay between chemokine receptors CXCR4 and CXCR2 and their ligands [67]. CXCR4 receptor regulates neutrophil homing in the bone marrow. Higher levels of CXCR4 and its ligands (for instance, CXCL12 also known as stromal cell-derived factor-1 (SDF-1), produced within the bone marrow) will restrain the neutrophils mobility, maintaining the neutrophil within its hematopoietic niche [68,69]. An initial step for neutrophil movement is the disruption of CXCR4 and its ligand expression by factors produced by tumors, including granulocyte-colony stimulating factor (G-CFS) [70]. Conversely, the CXCR2 receptor is responsible for neutrophils release into the circulation: CXCR2, CXCR2-ligands (CXCL1-3 and CXCL5-8), and G-CSF co-ordinate together to facilitate neutrophil mobilization from the bone marrow [68,69,70,71]. The mobilization of neutrophils to the tumor sites also requires an interplay between CXCR2 and its ligands CXCL1-3 and CXCL5-8 [72,73]. In cancer, the CXCR2 axis is the primary player for neutrophil recruitment to the tumor sites [74] combined with G-CSF [68,70]. Multiple cell types within the tumor produce the CXCR2 chemokines including tumor cells, immune cells, and cancer-associated fibroblasts [73,75,76].

The feasibility of using neutrophils to improve cancer therapy has been shown in a mouse melanoma model [77]. Systemically delivered albumin-NPs, loaded with pyropheophorbide-a, accumulate in melanoma lung metastases when a monoclonal antibody specific for gp75 antigen of melanoma (anti-gp75 mAb TA99) is injected in the melanoma-bearing animals. In the presence of the antibody, albumin-NPs hijack circulating neutrophils which accumulate within the tumor and the photodynamic therapy suppresses tumor growth. This work provides a nice example of combining immunotherapy and nanotechnology. In tumor vessels there is a particular cooperation between platelets and neutrophils, which occurs at the interfaces between vessels and metastatic niches where hetero-aggregates of neutrophils and platelets promote neutrophil NET-osis [78], a particular feature of neutrophil defense against pathogens triggered by P-selectin expressed on platelets, through binding to its ligand PSGL-1 on neutrophils [79]. Neutrophil extracellular Traps (NETS) are the product of decondensed chromatin, structured as fibers composed by nuclear, cytoplasmic, and mitochondrial constituents. NETS are “sticky” and are therefore exploitable to entrap nano-sized therapeutics with high efficiency. Unfortunately, available data show the presence of very few NETs in the neutrophil-rich area of B16 melanoma cells experimental tumors in mice [80]. Neutrophils have a life span of about 7 days in vivo, that is reduced to a few hours in vitro, making difficult a quantitative preparation for tumor cell therapy (Table 1).

### 3.3. Monocytes/Macrophages (Mϕs)

Circulating monocytes have emerged as regulators of cancer development with various subsets that play opposite roles in promoting tumor growth or preventing metastases. Indeed, monocytes are the source of tumor-associated M*ϕ*s (TAMs) and dendritic cells (DCs), with different properties in shaping the pro- or anti-inflammatory cancer microenvironment [81]. The presence of the CCR2 chemokine receptor on the monocytes surface allows their recruitment within tumors by the monocyte chemoattractant protein-1 (CCL2), a chemokine which specifically mediates monocyte chemotaxis. These are properties to be exploited for a cell delivery system. As implied by their name, monocytes/M*ϕ*s can internalize considerable drug payloads with an expected excellent therapeutic effect. Upon recruitment into the tumor monocytes differentiate into M*ϕ*s, referred to as TAMs. TAMs migrate toward the hypoxic regions of the tumor where they discharge their NP payload. For this reason, monocytes have been widely studied as vehicles of nano-based therapeutics. Several approaches have been used: photothermal therapy with SPIONs to treat experimental pancreatic cancer [82], or with Au-silica-nanoshelles for breast cancer [83]. A severe direct cellular auto-toxicity, discouraging a therapeutic use, was observed upon loading or anchoring chemotherapeutic agents such as pegylated liposomal doxorubicin in monocytes/M*ϕ*s [84]. An interesting approach, which seems to be able to bypass the toxic effects of nanotherapeutic products on “Trojan horse” macrophages, exploits the intrinsic phagocytic properties of TAMs. Miller et al. [85] have prepared therapeutic NPs comprising a fluorescent platinum(IV) pro-drug and a clinically tested polymer platform (PLGA-b-PEG) that promote long drug circulation and accumulation by directing cellular uptake toward tumor-associated macrophages (TAMs). TAMs internalize and release therapeutic platinum to experimental brain metastases of fibrosarcoma, ovarian carcinoma, and murine lung tumors. 

Taking advantage of the high affinity of hyaluronic acid (HA) for CD44 receptors of monocytes/M*ϕ*s it is possible to construct cell-based bio-hybrid devices adherent to the monocyte cell surface that do not interfere with cell functionality. Such surface cargos (“backpackages”) are composed by multi-layers of bio-compatible materials that leverage the functions of encapsulated cargo (NPs, drugs) and the innate tumor-homing capabilities of the cell, with the possibility to release the cargo in a controlled manner [86]. However, even if these approaches overcome the limitations of EPR-based drug delivery systems for cancer treatment, the off-target monocytes localization may severely hamper their clinical translation, as shown by liver, spleen, and lung sequestration [87,88]. A preclinical study reports the use of monocytes/Mϕ in suicide cytotherapy of melanoma, showing that NP-dependent prodrug (irinotecane) loading did not improve the therapeutic effect [89]. Monocytes make up between 3% and 8% of the circulating cell population. The life span of a circulating monocyte is fairly brief (from hours to days) and most undergo apoptosis in about 24 h. The life span of circulating M*ϕ*s is 1-3 days, while tissue-resident M*ϕ*s may survive for years. However, monocytes can be easily prepared from the same patient, loaded in vitro with NPs, and reintroduced into the patient, where they have a long life-span once within the target tumor (Table 1).

### 3.4. Lymphocytes

The use of lymphocytes as NP-delivery cell system in tumors has been previously reviewed, highlighting how the CXCR4-CCL12 axis is the most used by all classes of T lymphocytes for homing in tumors and in the bone marrow [90]. Nanoparticles can be either incorporated or surface-immobilized on leukocytes in a “hitchhiking strategy.” By attaching NPs drug cargos on the surface of cytotoxic T lymphocytes (CTL), Jones et al. [91] have obtained an antigen-triggered release following antigen recognition, an interesting system which could be useful in cancer therapy. In this system, the killing components of cytotoxic lymphocytes, lytic granules, and perforin, served as triggers to release therapeutic payloads from CTL-attached nanoscale lipid nanocapsules containing immunotherapeutic agent to an anatomical site of HIV viral replication. One out of the several limiting factors of chimeric antigen receptor (CAR) T-cell therapy for treatment of solid cancers, including melanoma, is the production of immunosuppressive molecules, such as adenosine, in the tumor microenvironment (TME). Upon binding to its receptor (A2aR) on CD4^+^ and CD8^+^ T cells, adenosine inhibits the lymphocyte function. This suppression can be blocked by the A2aR-specific antagonist SCH-58261 (SCH), but its applications have been limited by the difficulty of delivering the antagonist to immune cells present in the TME. To solve the problem Siriwon et al. [92] have used engineered CAR-T as chaperones to deliver SCH-loaded cross-linked, multilamellar liposomal vesicles (cMLV) to tumor-infiltrating T cells in the immune-suppressive TME. This approach enhanced the efficacy of CAR T-cell therapy. cMLV nanoparticles covalent attachment to CAR-T cells did not affect their viability and effector functions. B cells have a life span from 4 days up to 5 weeks, while T cells may last from month to years (Table 1).

### 3.5. Red Blood Cells

The use of red blood cells (RBCs) in cell-based drug/NPs delivery has been recently reviewed [14]. Since circulating RBCs are unable to perform phagocytosis, hypotonic solutions are used to promote transient pores formation that allow therapeutic molecules to enter the cytoplasm and to be delivered throughout the circulatory system. RBCs have been investigated also for hitchhiking applications by attaching NPs to their surface, with the possibility to incorporate in the NPs the controlled mechanism for drug release [93,94,95]. Drug-loaded NPs and SPIONs can be incorporated within RBs taking advantage of the ex-vivo hypotonic pore-forming method. Since RBCs have not an innate tumor tropism, the tumor targeting may be obtained by application of magnetic fields and the activation/release of their NPs payload is achieved as a response to external commands such as light, heat, magnetic fields, ultrasounds [14]. Magnetic nanocarriers may help not only for a targeted delivery of chemotherapeutic agents into the tumor site but also provide contrast enhancing properties for diagnostics using magnetic resonance imaging (MRI), as reported in a study on experimental glioblastoma [96]. As detailed in a recent review [97], the RBCs membrane is also used to coat nanotherapeutics thus enhancing their stealthy properties as discussed above for platelets (“ghost-cell-strategy”). Wang et al. [98] fused RBC membranes and melanoma cell (B16-F10) membranes (RBC-B16) to coat DOX-loaded hollow copper sulfide NPs (DCuS@[RBC-B16]-NPs), which showed potential for synergistic PTT/chemotherapy of melanoma. Red blood cells-membrane-enveloped polymeric nanoparticles have also been used as nanovaccine for the induction of antitumor immunity against melanoma. [99] (Table 1).

### 3.6. Neural Stem Cells and Induced Pluripotent Stem Cells

Stem cells have an extraordinary innate tumor tropism, however both neuronal stem cells (NSCs) and induced pluripotent stem cells (iPSCs) present important disadvantages. NSCs are difficult to prepare because the adult brain harbors a small amount of dormant cells [100]. Therefore, transduction of normal fibroblasts with lentiviral vectors encoding the transcription factors Brn2, Sox2, *and* FoxG1 is used to prepare induced neural stem cells (iNSCs), that have been used to cross the blood brain barrier to deliver drugs for brain malignancies (glioblastoma) and neurodegenerative disorders [101]. Rachakatla and coworkers [102] developed aminosiloxane-porphyrin-functionalized magnetic NPs and transplanted neural progenitor cells (NPCs) loaded with this cargo into mice with melanoma. The targeted delivery of MNPs by the cells resulted in a measurable regression of the tumors. Both NSCs and iNSCs show properties similar to mesenchymal stem cells (MSCs), including the property to be recruited by the CXCR4/SDF-1 axis [103,104], so that stem cell treatment to deliver drugs to neural tumors by iNSC is currently under clinical trial. iPSCs [105] have raised serious concerns related to their potential to give origin to malignant teratomas following in vivo transplantation [106] (Table 1).

### 3.7. Mesenchymal Stem Cells

No alarm for safety has been described for the use of MSCs. They do not form tumors and drug-engineered MSCs may be rapidly prepared for rapid transplantation from bone marrow [107] and from pieces of the umbilical cord walls [108]. MSCs have a remarkable expansion potential in culture and are prone to genetic modifications with viral vectors, thus providing optimal delivery vehicles for cell-based gene therapy. MSCs are attracted within tumors by at least two mechanisms: the CXCR4/SDF-1(CXCL12-chemokine) axis [109] and CXCR4/MIF (migration inhibiting factor) axis [110]. The role of SDF-1 in MSC homing to tumor cells, however, is disputed [111]. Factors secreted from tumor cells can trigger SDF-1 secretion from MSCs, activating their motility [109], but competing with tumor-produced SDF-1 for recruitment of circulating therapeutic MSC. MIF expression in tumors closely correlates with their aggressiveness and metastatic potential [112,113,114,115]. CXCR4/MIF is the dominant chemotactic axis in MSC recruitment to tumors [110]. On these basis, MSCs have been used to inhibit tumor angiogenesis [116] and tumorigenesis [117], as well as therapeutic cytoreagents for tumor gene therapy [118]. MSCs have been used in suicide gene therapy, an approach based on arming tumor-associated cells with viral vectors expressing genes which produce enzymes able to metabolize prodrugs into cancer drugs that kill the tumor cells by a “bystander effect” [119]. MSCs act as immunostimulants in the tumor microenvironment [120] and their immunomodulating properties have been recently reviewed [121]. Further, MSCs have been used as carriers of oncolytic adenovirus resulting in enhanced oncolytic virotherapy [122]. The MSC-mediated oncolytic approach has been used also in experimental melanoma [123] and the potential of MSCs to deliver targeted agents in experimental melanoma has been previously reviewed [124]. An excellent survey of the use of NP-based therapeutics for melanoma treatment does not take in consideration MSCs or other cell-mediated delivery systems [125]. In the light of the strong evidence of magnetic resonance imaging of pulmonary metastases with magnetic NPs/ MSCs [126], tumor targeting with silica NPs/MSCs [127] and photothermal therapy with gold NPs/MSCs [128], it is our opinion that the theranostic use of MSC/NPs in melanoma is near to cross the boundary between the preclinical and the clinical phase.

Actually, monocytes/Mϕ and autologous and allogeneic MSC are the most used cells in cell-delivered AuNPs for treatment of a wide range of clinical diseases [15]. Because of their ease of preparation from cord blood, allogeneic MSCs are especially attractive because of their immediate availability and care at the time of disease diagnosis. However, studies that control for MHC expression have reported both cell-mediated and humoral immune responses to MHC-mismatched MSCs [129] (Table 1).

### 3.8. Endothelial Colony Forming Cells (ECFCs)

The acronym EPCs (endothelial progenitor cells) encompasses a series of multiple early and late progenitors of mature endothelial cells, currently used in vessel repair or regeneration therapies [130]. A Consensus Statement on Nomenclature of endothelial progenitors has recommended the term “ECFCs” (Endothelial Colonies Forming Cells), discouraging the current generical term “EPCs” [131]. The same authors have proposed a characterization of ECFCs based on a defined cellular phenotype and function [132]. ECFCs are adult endothelial progenitor cells that have high proliferative potential and are capable of differentiating to regenerate endothelial cell populations [133]. ECFCs express endothelial markers CD31, CD146, VEGFR2, vWF, and VE-cadherin. They also express CD34, a marker that diminishes as the cells are expanded in vitro. ECFCs are negative for hematopoietic markers CD14 and CD45. The main characteristic of ECFCs is their ability to form a de novo vascular network in vitro while in vivo they integrate with the host vasculature [133,134,135,136,137]. On these basis, all the previous abundant literature using “late EPCs” must be related to *bona fide* ECFCs. Most importantly, ECFCs are actively recruited in tumors [16,138], including melanoma [139,140]. We have shown that melanoma cells recruit Au-NPs-loaded ECFCs prepared from cord blood (CB) by the CXCR4 (expressed by ECFCs)-SDF-1 (produced by melanoma cells and associated MSCs) axis in 18 h, and that an anti-SDF-1 neutralizing antibody precludes ECFCs recruitment both in vitro and in vivo [140]. The strength of the interaction allows to identify Au-NPs-ECFCs in the tumor mass even after 4 weeks from the injection, while they disappear from normal organs (kidneys, lung, liver) in about 24 h. Such differential times allow a theranostic approach with diagnosis obtained by photoacoustic imaging (PAI) and thermoablation by PAI-guided laser therapy. It appears that CB stem cells, including ECFCs, are relatively tolerant and are less likely to react immunologically against the host, with lower incidence and less severe graft-versus-host disease with allogeneic grafts than with bone marrow and peripheral blood, despite the HLA disparity [129], thereby providing higher chances of finding a donor for populations who are under-represented in donor registries. With the current availability of about 100 million per year, CB is the largest source of stem cells available and to date more than 450,000 units are banked worldwide and are available for potential clinical use [141]. Finally, the thermoablative and diagnostic properties of each cell type containing a payload of NPs are obviously linked to the quantity of NPs that each cell type is able to incorporate and therefore to convey within the neoplastic mass. We have performed multiple measures of AuNPs uptake by ECFCs, Mϕ, and MSCs. Summarizing the results of analyses, Mϕ internalize particles better than MSCs but in a largely lower quantity than the ECFCs. (Table 1).

## 4. Nanoparticles in the Preclinical Research and Clinic of Melanoma

Recent reviews have highlighted the available NP-based therapeutics for the treatment and imaging of melanoma. In a survey of 2018 Bagheri et al. [10] listed a series of seven preclinical studies using gold NPs, ranging from photoacoustic detection to thermal destruction of malignant cells and improving radioresponses of melanoma cells. Mishra et al. [9] provided a complete list of 32 preclinical contributions using all the available NP-based “organic soft nanocarriers” (as amphiphilic polymers and liposomes) and of “hard NPs” (quantum dots, gold and mesoporous silica particles), with only a single contribution on melanoma. An update on available NP-therapeutics in the clinic [142] shows that there are four products potentially available for therapy of melanoma. Abraxane (Celgene), an albumin-particle bound paclitaxel, FDA-approved for advanced non-small cell lung cancer, metastatic breast cancer, and metastatic pancreatic cancer, is actually under investigation also in melanoma patients. Marquibo (Spectrum), a non-PEGylated liposomal vincristine is investigated for uveal melanoma therapy and FDA-approved for Philadelphia chromosome-negative lymphoblastic leukemia. Cornell-Dots, silica nanoparticles with a NIR (near-infrared) fluorophore, PEG coating and a ^124^I-radiolabeled cRGDY-targeting peptide are under clinical trial for imaging of melanoma and malignant brain tumors. Lipo-MERIT (Biontech RNA Pharmaceuticals), a liposome formulation containing four RNA-dryg products, investigated as a cancer vaccine for advanced melanoma, was still in the recruiting phase in 2019.

## 5. Conclusions

In view of the amount of preclinical work that is underway with the use of cell-delivered nanoparticles one could be tempted to anticipate that we are indeed “on the precipice of a drug delivery revolution” [14]. However, melanoma, as well as many other tumors, suffers of a paucity of research and of proposed clinical applications in this advanced field, which may be related to high manufacturing costs and to the multidisciplinary approaches required to realize an efficient final product. According to the Ockham’s razor principle, if there are two or more explanations or solutions for an occurrence, the one that requires the smallest number of assumptions is usually correct. Cell-mediated delivery requires: (1) Loading of nanoparticles, which is easily achievable by exploiting ex vivo phagocytosis of hard quantum dots, gold, and silica particles by phagocytosis-prone cells with a high cytoplasm/nuclear ratio; (2) a marked tumor tropism and quickly leave of healthy organs; (3) traceability with simple imaging methods; (4) ease of stimulation to produce locally controllable destructive energy for tumor mass without affecting normal surrounding tissues. We are aware that respecting these few parameters is absolutely not simple, but what we know about mesenchymal stem cells and endothelial colony forming cells-loaded NPs, authorizes us to hope for a prompt awareness by the scientific community. This kind of approach would provide controlled dosing, no systemic toxicity, and high tolerance by the patient.

*“What the medicine does not cure, the scalpel heals, what the scalpel does not heal, the fire heals it, what fire does not heal must be considered incurable”*

Hippocrates (460–377 B.C)

## Figures and Tables

**Table 1:** Main features of cell-based nanoparticle delivery systems in cancer therapy.

Cell Type	Delivery System	Therapeutic Mechanism	Target	Tolerability	Life Span	Main Drawabacks	Ref.
**Platlets**	Membrane-coated gold nanostars containing curcumin (ghost-cells)	NIR Controlled release	Melanoma primary tumor; Possible metastasis	YES (source: autologous blood)	7-–10 days	No proliferation; Issues with purification;	[40,41,42,43,44,45,46,47,48,49,50,51,52,53,54,55,56,57]
**Neutrophils**	Albumin-NPs loaded with pyropheophorbide-a, and anti-GP75 mAb	Photodynamic therapy	Melanoma primary tumor; Metastasis	Yes (source: autologous blood)	7 days in vivo Few hours in vitro	Short life	[58,59,60,61,62,63,64,65,66,67,68,69,70,71,72,73,74,75,76,77,78,79,80]
**Monocytes**	SPIONs- or Au nanoshells-loaded; liposome-doxorubicin-loaded	Hyperthermia; Release of encapsulated therapeutic cargos	Pancreatic cancer; breast cancer; experimental lung metastases of melanoma	Yes (source: autologous blood)	1–3 days for circulating cells; years for tissue-resident Mϕ	Liver, spleen and lungs sequestration; direct toxic effects of NP-chemotherapeutic cargos	[81,82,83,84,85,86,87,88,89]
**Lymphocytes**	Internal or surface-immobilized NP systemic delivery of engineered CAR-T cross-linked to multilamellar liposomal vescicles (cMLV) containing a specific inhibitor (SHC) of immunosuppressor A2aR	Inhibition of the tumor immunosuppressive microenvironment.	Experimental human ovarian cancer; Experimental chronic myelogenous leukemia; Possible metastasis	Yes	4 days–5 weeks for B cells, months -years for T cells	Acute anaphylaxis; tumor lysis syndrome (TLS); cytokine release syndrome (CRS)	[90,91,92]
**Red blood cells**	Loaded with SPIONs in hypotonic solutions; hijacked with SPIONs or polymeric NP; DOX-loaded hollow copper sulfide NPs coated with RBC and melanoma cell membranes; enveloped polymeric nanoplatform	Photothermal therapy (PTT); Chemotherapy; Anti-tumor immunity	Glioblastoma; melanoma;	Yes (source: autologous blood)	3months	None	[14], [93,94,95,96,97,98,99]
**NSCs or iPSCs**	Loaded with aminosiloxane-porphyrin functionalized magnetic NPs with core/shell Fe/Fe(3)O	Magnetic hyperthermia	Primary melanoma	Yes (NCS: autologous origin); (iPSC:Low immunogenicity)	Self renewing when cultured and expanded	NSC: difficult to prepare; iPSC, potential induction of teratomas after in vivo transplant.	[100,101,102,103,104,105,106]
**MSCs**	Loaded with SPIONs, silica or Au-NP	Magnetic or plasmonic hyperthermia	Breast cancer; glioblastoma; human fibrosarcoma;	Yes: autologous or allogeneic origin , prepared from the wall of umbilical cord vessels	Self renewing when cultured and expanded	Cell-mediated and humoral immune responses to MHC-mismatched MSC	[107,108,109,110,111,112,113,114,115,116,117,118,119,120,121,122,123,124,125,126,127,128,129]
**ECFCs**	Labelled with 111-In loaded with Au-NP	Phothermal therapy	Primary melanoma	Yes: autologous or allogeneic origin, prepared from umbilical cord blood	Self renewing when cultured and expanded	No major cell-mediated and humoral immune responses to MHC-mismatched MSC	[130,131,132,133,134,135,136,137,138,139,140,141]

Abbreviations: NSCs, neural stem cells; iPSCs, induced pluripotent stem cells; MSCs, mesenchymal stem cells; ECFCs, endothelial colony forming cells; SPIONs, superparamagnetic iron oxide nanoparticles; Au-nanoshells; gold nanoshell; CAR-T, chimeric antigen receptor T-cell; NIR, near infrared.
